# Supplementary material for: Environmental Drivers of Ranavirus in Free-Living Amphibians in Constructed Ponds
Source: Ecohealth. 2018 Aug 9;15(3):608–18. doi: 10.1007/s10393-018-1350-5 (PMC6245063; doi:10.1007/s10393-018-1350-5)
Supplement: Supplementary file 1 — Supplementary material 1 (DOCX 160 kb) [file 10393_2018_1350_MOESM1_ESM.docx]

# Supplementary Materials for the manuscript

# Environmental drivers of a widespread ranavirus in free living amphibians in constructed ponds

**Supplementary Methods - Quantitative PCR protocol**

Negative or ambiguous results were analyzed via quantitative PCR at Cornell University Animal Health Diagnostic Center using the following protocol modified from Pallister et al. (2007): Five μL template DNA was added to 5 μL Invitrogen TaqMan® Fast Virus 1-Step Master Mix, 0.05 μL fluorescent probe (100 μM; 5'-CAC AAC ATT ATC CGC ATC-3'), and 0.18 μL primers (100 μM; rtMCP-F: 5'-CTC ATC GTT CTG GCC ATC AA-3'; rtMCP-R: 5'-TCC CAT CGA GCC GTT CA-3') to a total volume of 20 μL. Samples were run alongside negative and positive controls in 48-well plates using Applied Biosystems StepOne™ real-time PCR system and analyzed with StepOne software v2.3. A synthetic Ultramer® oligomer containing binding sites from primers and probe described above was used as positive control (R. Ossiboff, Cornell University Animal Health Diagnostic Center; 5'- AAG ACT TGG CCA CTT ATG ACT TGC ATC GGC AGC AAA TCT CAT CGT TCT GGC CAT CAA CCA CAA CAT TAT CCG CAT CAT CAA CGG CTC GAT GGG ATG CCA TAT TTT AAG AGA ATT ATC GAG GTC TCT GGA GAA CAA GAA CG - 3'). Five serial dilutions of 1:10 were run in duplicate and used to calibrate a set of standards, and a cycle threshold (C_T_) was set at the logarithmic center of standard linear growth curves for each run. Samples with C_T_ < 36 were declared positive; this threshold was based on mean C_T_ values of the lowest concentration of positive control (1 x 10^3^ nM).

Supplementary Figure – Predictor variables

Figure S1: No strong covariation was observed among the predictor variables used in the generalized linear models of Frog virus 3 prevalence.


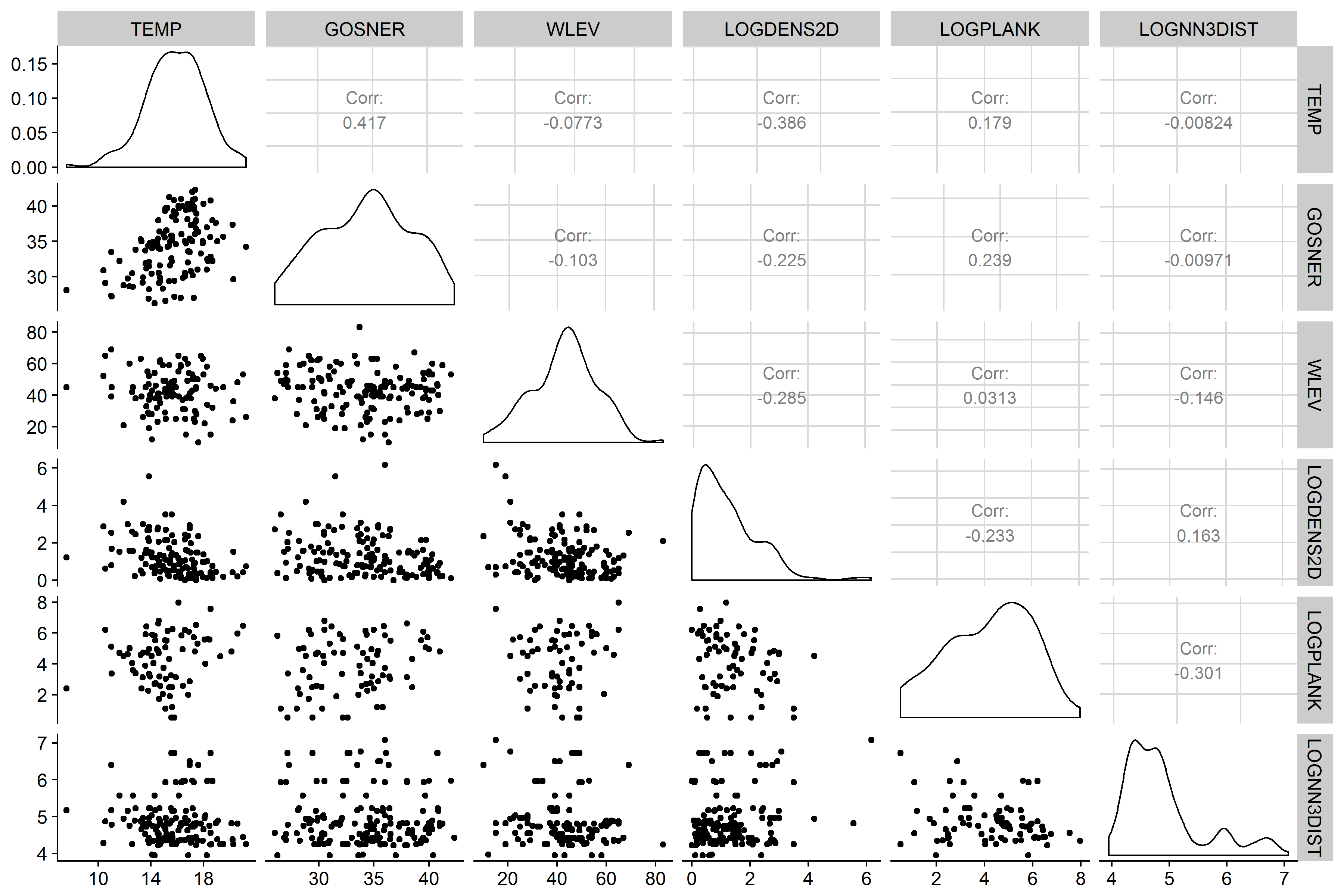


Supplementary Table – Model selection

Table S1: Model selection results for generalized linear models of Frog virus 3 prevalence. IC_LOO_: Leave-one-out cross validation Information Criterion; p_LOO_: estimated effective number of parameters; Δ_IC_ IC_LOO_ difference to best model; SE() standard error. See Table 1 for predictor variable descriptions. The cross validation procedure yields an estimate of the information criterion, and the associated uncertainty. Model comparison is therefore based not just on Δ_IC_ values (and arbitrary difference thresholds), as is routinely done with AIC differences in maximum likelihood frameworks, but by taking the standard error for the estimated Δ_IC_ into account.

| Model | IC_LOO_ | SE(IC_LOO_) | Δ_IC_ (SE) | p_LOO_ | SE(p_LOO_) |
| --- | --- | --- | --- | --- | --- |
| TEMP + GOSNER + log(DENS) + WLEV + log(PLANK) + log(DIST) | 704 | 65 |  | 117.4 | 15.4 |
| TEMP + GOSNER + log(DENS) + WLEV + log(PLANK) | 712 | 67 | 8 (18) | 113.2 | 14.7 |
| TEMP + GOSNER + log(DENS) + WLEV + log(DIST) | 989 | 116 | 286 (85) | 115.5 | 22.1 |
| TEMP + GOSNER + log(DENS) + WLEV | 1003 | 112 | 300 (84) | 116.8 | 22.5 |

**References**

Pallister J, Gould A, Harrison D, Hyatt A, Jancovich J, Heine H. 2007. Development of real-time PCR assays for the detection and differentiation of Australian and European ranaviruses. J. Fish Dis. 30:427–438.
